# Supplementary material for: Random forest machine-learning algorithm classifies white- and brown-rot fungi according to the number of the genes encoding Carbohydrate-Active enZyme families
Source: Appl Environ Microbiol. 2024 Jun 4;90(7):e00482-24. doi: 10.1128/aem.00482-24 (PMC11267879; doi:10.1128/aem.00482-24)
Supplement: Supplemental material — Tables S1 and S2; Fig. S1 to S4. [file aem.00482-24-s0001.pdf]

Supplementary materials for

Random forest machine-learning algorithm classifies white- and brown-rot fungi according to the number of the genes encoding Carbohydrate-Active enZyme families

Natsuki Hasegawa, Masashi Sugiyama and Kiyohiko Igarashi

Table S1 List of fungi used for the analysis with abbreviations and decay modes

Table S2 Gini importance of all CAZymes in RF models predicting decay modes

Figure S1 Consensus tree.

Figure S2 Choice of oversampling technique

Figure S3 Distribution of genes by decay mode (families ranked 13-30 in importance)

Figure S4 Estimated class probabilities (families ranked 13-30 in importance)

Table S1 List of fungi used for the analysis with abbreviations and decay modes

MycoCosm	Project code	Order	Decay modes
Leratiomyces ceres mycota028 v1.0	Lerce1	Agaricales	Saprotroph
Leratiomyces erythrocephalus mycota031 v1.0	Lerer1	Agaricales	Saprotroph
Hypholoma sublateritium v1.0	Hypsu1	Agaricales	White-rot
Pholiota conissans CIRM-BRFM 674 v1.0	Phocon1	Agaricales	Litter-decomposing
Pholiota molesta CBS 144467 v1.0	Phohig1	Agaricales	White-rot
Pholiota alnicola AH 47727 v1.0	Phoaln1	Agaricales	White-rot
Hebeloma cylindrosporum h7 v2.0	Hebcy2	Agaricales	Ectomycorrhizal
Hebeloma brunneifolium PMI1Jessy v2.0	Hebvel2	Agaricales	Ectomycorrhizal
Psilocybe cubensis v1.0	Psicub1_1	Agaricales	Saprotroph
Psilocybe serbica v1.0	Psiser1	Agaricales	Saprotroph
Agrocybe pediades AH 40210 v1.0	Agrped1	Agaricales	Litter-decomposing
Agrocybe praecox OKM6292 v1.0	Agrpra2	Agaricales	Litter-decomposing
Gymnopilus chrysopellus PR-1187 v1.0	Gymch1	Agaricales	White-rot
Gymnopilus junonius AH 44721 v1.0	Gymjun1	Agaricales	White-rot
Galerina marginata v1.0	Galma1	Agaricales	White-rot
Crepidotus cesatii CBS 511.95 v1.0	Creces1	Agaricales	White-rot
Crepidotus variabilis CBS 506.95 v1.0	Crevar1	Agaricales	White-rot
Phaeosolenia platensis CBS 268.64 v1.0	Phapla1	Agaricales	Saprotroph
Cortinarius austrovenetus TL2843-KIS7R v1.0	Coraus1	Agaricales	Ectomycorrhizal
Cortinarius sp. KIS3-TL2766 v1.0	CorKIS3_1	Agaricales	Ectomycorrhizal
Cortinarius aff. *campbellae* TAS5-PSC4363 v1.0	Corcam1	Agaricales	Ectomycorrhizal
Cortinarius glaucopus AT 2004 276 v2.0	Corgl3	Agaricales	Ectomycorrhizal
Crassisporium funariophilum CBS 144457 v1.0	Crafun1	Agaricales	Saprotroph
Bolbitius vitellinus SZMC-NL-1974 v1.0	Bolvit1	Agaricales	Litter-decomposing
Conocybe apala SZMC-NL-8967 v1.0	Conapa1	Agaricales	Litter-decomposing
Panaeolus papilionaceus CIRM-BRFM 715 v1.0	Panpap1	Agaricales	Saprotroph
Coprinopsis cinerea	Copci1	Agaricales	Saprotroph
Coprinopsis cinerea AmutBmut pab1-1 v1.0	Copci_AmutBmut1	Agaricales	Saprotroph
Coprinopsis sp. MPI-PUGE-AT-0042 v1.0	Copph3	Agaricales	Saprotroph
Coprinopsis sclerotiger v1.0	Copscl1	Agaricales	Saprotroph
Coprinopsis marcescibilis CBS121175 v1.0	Copmar1	Agaricales	Saprotroph
Coprinellus angulatus CBS 144469 v1.0	Copang1	Agaricales	Saprotroph
Coprinellus pellucidus v1.0	Coppel1	Agaricales	Saprotroph
Coprinellus micaceus FP101781 v2.0	Copmic2	Agaricales	Saprotroph
Laccaria bicolor S238N-H82 v1.0	LacbiH82_1	Agaricales	Ectomycorrhizal
Laccaria bicolor S238N-H82xH70 v1.0	LacbiH82xH70_1	Agaricales	Ectomycorrhizal
Laccaria bicolor S238N-H53 v1.0	LacbiH53_1	Agaricales	Ectomycorrhizal
Laccaria bicolor S238N 93.12 v1.1	Lacbi9312_2	Agaricales	Ectomycorrhizal
Laccaria bicolor S238N-H70 v1.0	LacbiH70_1	Agaricales	Ectomycorrhizal
Laccaria bicolor S238N v1.0	LacbiS238N_1	Agaricales	Ectomycorrhizal
Laccaria bicolor DR170 v1.0	LacbiDR170_1	Agaricales	Ectomycorrhizal
Laccaria bicolor S238O v1.0	LacbiS238O_1	Agaricales	Ectomycorrhizal
Laccaria bicolor CBS 559.96 v1.0	Lacbi55996_1	Agaricales	Ectomycorrhizal

Laccaria bicolor Cham3 v1.0	LacbiCham3_1	Agaricales	Ectomycorrhizal
Laccaria bicolor CBS 594.89 v1.0	Lacbi59489_1	Agaricales	Ectomycorrhizal
Laccaria bicolor N203 v1.0	LacbiN203_1	Agaricales	Ectomycorrhizal
Laccaria bicolor 81306 v1.0	Lacbi81306_1	Agaricales	Ectomycorrhizal
Laccaria bicolor D101 v1.0	LacbiD101_1	Agaricales	Ectomycorrhizal
Laccaria amethystina LaAM-08-1 v2.0	Lacam2	Agaricales	Ectomycorrhizal
Crucibulum laeve CBS 166.37 v1.0	Crula1	Agaricales	White-rot
Cyathus striatus AH 40144 v1.0	Cyastr2	Agaricales	White-rot
Leucoagaricus gongylophorus AB2 v1.0	Leugon1	Agaricales	Insect-symbiotroph
Leucoagaricus gongylophorus AS2 v1.0	LeugonAS2_1	Agaricales	Insect-symbiotroph
Leucoagaricus gongylophorus Ac12	Leugo1_1	Agaricales	Insect-symbiotroph
Leucoagaricus gongylophorus AL2 v1.0	LeugonAL2_1	Agaricales	Insect-symbiotroph
Lycoperdon perlatum FP-102459-T v1.0	Lycper1	Agaricales	Litter-decomposing
Macrolepiota fuliginosa MF-IS2 v1.0	Macfu1	Agaricales	Litter-decomposing
Agaricus bisporus var. *burnettii* JB137-S8	Agabi_varbur_1	Agaricales	Litter-decomposing
Agaricus bisporus var *bisporus* (H97) v2.0	Agabi_varbisH97_2	Agaricales	Litter-decomposing
Amanita muscaria var. *formosa* 2016PMI152 v1.0	Amaapr1	Agaricales	Ectomycorrhizal
Amanita muscaria Koide v1.0	Amamu1	Agaricales	Ectomycorrhizal
Amanita rubescens Prilba v1.0	Amarub1	Agaricales	Ectomycorrhizal
Amanita thiersii Skay4041 v1.0	Amath1	Agaricales	Litter-decomposing
Pluteus cervinus NL-1719 v1.0	Plucer1	Agaricales	White-rot
Volvariella volvacea V23	Volvo1	Agaricales	White-rot
Tricholoma matsutake 945 v3.0	Trima3	Agaricales	Ectomycorrhizal
Tricholoma populinum 2016PMI031 v1.0	Tripop1	Agaricales	Ectomycorrhizal
Clitocybe sp. CONT 1119283 v1.0	Cont1119283	Agaricales	Saprotroph
Lepista nuda CBS 247.69 v1.0	Lepnud1	Agaricales	Litter-decomposing
Lyophyllum atratum CBS 144462 v1.0	Lyoat1	Agaricales	Ectomycorrhizal
Clitocybe gibba IJFM A808 v1.0	Cligib1	Agaricales	Litter-decomposing
Mycena haematopus CBHHK189 v1.0	Mychae1	Agaricales	Litter-decomposing
Mycena sanguinolenta CBHHK176m v1.0	Mycsan1	Agaricales	Litter-decomposing
Mycena galopus ATCC-62051 v1.0	Mycgal1	Agaricales	Litter-decomposing
Mycena albidolilacea CBHHK002 v1.0	Mycalb1	Agaricales	Litter-decomposing
Mycena olivaceomarginata CBHHK47/15 v1.0	Mycoli1	Agaricales	Litter-decomposing
Mycena polygramma CBHHK137 v1.0	Mycpol1	Agaricales	Litter-decomposing
Mycena vitilis CBHHK169m v1.0	Mycvit1	Agaricales	Litter-decomposing
Mycena capillaripes Frankland 9286 v1.0	Mycrub1	Agaricales	Litter-decomposing
Mycena leptocephala CBHHK5/15 v1.0	Myclep1	Agaricales	Litter-decomposing
Mycena alexandri CBHHK200 v1.0	Mycale1	Agaricales	Litter-decomposing
Mycena metata CBHHK182m v1.0	Mycmet1	Agaricales	Litter-decomposing
Mycena filopes CBHHK001 v1.0	Mycfil1	Agaricales	Litter-decomposing
Mycena pura 9144 v1.0	Mycpur1	Agaricales	Ectomycorrhizal
Mycena amicta BAPX v1.0	Mycami1	Agaricales	Litter-decomposing
Mycena crocata CBHHK184 v1.0	Myccro1	Agaricales	Litter-decomposing
Panellus stipticus KUC8834 v1.1	Panst_KUC8834_1_1	Agaricales	White-rot
Panellus stipticus LUM v1.0	Panst_LUM_1_1	Agaricales	White-rot
Roridomyces roridus 9284 v1.0	Rorror1	Agaricales	Litter-decomposing

Mycena galericulata CBHHK162m v1.0	Mycgale1	Agaricales	Litter-decomposing
Mycena maculata CBHHK188m v1.0	Mycmac1	Agaricales	Litter-decomposing
Mycena latifolia 10383 v1.0	Myclat1	Agaricales	Litter-decomposing
Mycena belliae CBHHK173m v1.0	Mycbel1	Agaricales	Litter-decomposing
Mycena rosella CBHHK067 v1.0	Mycros1	Agaricales	Litter-decomposing
Mycena vulgaris CBHHK164 v1.0	Mycvul1	Agaricales	Litter-decomposing
Mycena epipterygia CBHHK145m v1.0	Mycepi1	Agaricales	Litter-decomposing
Mycena sp. CBHHK59/15 v1.0	Myc59_1	Agaricales	Litter-decomposing
Mycena rebaudengoi CBHHK068 v1.0	Mycreb1	Agaricales	Litter-decomposing
Lentinula edodes TMI1148 v1.0	LedTMI1148_1	Agaricales	White-rot
Lentinula edodes Le(Bin) 0899 ss11 v1.0	Lenedo1	Agaricales	White-rot
Lentinula edodes VB361 v1.0	LedVB361_1	Agaricales	White-rot
Lentinula edodes NBRC 111202	Lened1	Agaricales	White-rot
Lentinula edodes B17 v1.3	LedB17_3	Agaricales	White-rot
Lentinula edodes W1-26 v1.0	Lentinedodes1	Agaricales	White-rot
Lentinula edodes CS-584 v1.0	Led_CS584_1	Agaricales	White-rot
Lentinula edodes TMI1633 v1.0	LedTMI1633_1	Agaricales	White-rot
Lentinula afn. *lateritia* sp2 HRB7682 ss15 v1.0	Lenafn1	Agaricales	White-rot
Lentinula lateritia RHP3577 ss4 v1.0	Lenlat1	Agaricales	White-rot
Lentinula lateritia RV95-379 v1.0	LlaRV95_379_1	Agaricales	White-rot
Lentinula novae-zelandiae TMI-1172 v1.0	Lennov1	Agaricales	White-rot
Lentinula novae-zelandiae ICMP 18003 v1.0	LnoICMP18003A_1	Agaricales	White-rot
Lentinula afn. *lateritia* sp 3 TMI1502 v1.0	Lenafn_TMI1502_1	Agaricales	White-rot
Lentinula lateritia TMI1499 v1.0	LlaTMI1499_1	Agaricales	White-rot
Lentinula raphanica TFB8682 v1.0	Lenra_T_1	Agaricales	White-rot
Lentinula raphanica TFB9207 v1.0	LraTFB9207_1	Agaricales	White-rot
Lentinula raphanica JLM1587 v1.0	LraJLM1587_1	Agaricales	White-rot
Lentinula raphanica INPA1820 v1.0	LraINPA1820_1	Agaricales	White-rot
Lentinula raphanica TFB9929 ss4 v1.0	Lenrap1_155	Agaricales	White-rot
Lentinula raphanica INPA1701G ss19 v1.0	Lenrap1	Agaricales	White-rot
Lentinula boryana TFB7810 v1.0	LboTFB7810_1	Agaricales	White-rot
Lentinula afn. *boryana* TFB7829 v1.0	LboTFB7829_1	Agaricales	White-rot
Lentinula boryana ET3784 v1.0	LboET3784_1	Agaricales	White-rot
Lentinula boryana TFB10291 v1.0	Lbo_TFB10291_1	Agaricales	White-rot
Lentinula boryana TFB10292 v1.0	LboTFB10292_1	Agaricales	White-rot
Lentinula boryana TFB10827 v1.0	Lbo_TFB10827_1	Agaricales	White-rot
Rhodocollybia butyracea CCBAS 279 v1.0	Rhobu1	Agaricales	Litter-decomposing
Rhodocollybia butyracea AH 40177 v1.0	Rhobut1_1	Agaricales	Litter-decomposing
Gymnopus luxurians v1.0	Gymlu1	Agaricales	Saprotroph
Gymnopus androsaceus JB14 v1.0	Gyman1	Agaricales	Litter-decomposing
Gymnopus earleae GB-263.02 v1.0	Gymear1	Agaricales	Litter-decomposing
Omphalotus olearius	Ompol1	Agaricales	Saprotroph
Dendrothele bispora CBS 962.96 v1.0	Denbi1	Agaricales	White-rot
Moniliophthora perniciosa FA553	Monpe1_1	Agaricales	Saprotroph
Marasmius fiardii PR-910 v1.0	Marfi1	Agaricales	Litter-decomposing
Armillaria solidipes 28-4 v1.0	Armost1	Agaricales	White-rot

Armillaria ostoyae C18/9	Armosto1	Agaricales	White-rot
Armillaria borealis FPL87.14 v1.0	Armbor1	Agaricales	White-rot
Armillaria gallica 21-2 v1.0	Armga1	Agaricales	White-rot
Armillaria cepistipes B5	Armcep1	Agaricales	White-rot
Armillaria nabsnona CMW6904 v1.0	Armnab1	Agaricales	White-rot
Armillaria fumosa CBS 122221 v1.0	Armfum1	Agaricales	White-rot
Armillaria luteobubalina HWK02 v1.0	Armlut1	Agaricales	White-rot
Armillaria novae-zelandiae 2840 v1.0	Armnov1	Agaricales	Plant-pathogen
Armillaria mellea DSM 3731	Armme1_1	Agaricales	White-rot
Armillaria ectypa FPL83.16 v1.0	Armect1	Agaricales	White-rot
Armillaria tabescens CCBAS 213 v1.0	Armtab1	Agaricales	White-rot
Guyanagaster necrorhizus MCA 3950 v1.0	Guyne1	Agaricales	Unknown
Hymenopellis radicata IJFM A160 v1.0	Hymrad1	Agaricales	Saprotroph
Oudemansiella mucida CBS 558.79 v1.0	Oudmuc1	Agaricales	White-rot
Cylindrobasidium torrendii FP15055 v1.0	Cylto1	Agaricales	White-rot
Cystostereum murrayi CysMur001 v1.0	Cysmur1	Agaricales	White-rot
Mycena floridula CBHHK072 v1.0	Mycflo1	Agaricales	Litter-decomposing
Schizophyllum commune 223.1 v1.0	Schco2231_1	Agaricales	White-rot
Schizophyllum commune 225.1 v1.0	Schco2251_1	Agaricales	White-rot
Schizophyllum commune 227.1 v1.0	Schco2271_1	Agaricales	White-rot
Schizophyllum commune 207.1 v1.0	Schco2071_1	Agaricales	White-rot
Schizophyllum commune 227.2 v1.0	Schco2272_1	Agaricales	White-rot
Schizophyllum commune Loenen D v1.0	Schco_LoeD_1	Agaricales	White-rot
Schizophyllum commune ZB2 v1.0	SchcoZB2	Agaricales	White-rot
Schizophyllum commune Tattone D v1.0	Schco_TatD_1	Agaricales	White-rot
Schizophyllum commune ZB1 v1.0	SchcoZB1	Agaricales	White-rot
Schizophyllum radiatum CBS 301.32 v1.0	Schrad1	Agaricales	White-rot
Auriculariopsis ampla NL-1724 v1.0	Auramp1	Agaricales	White-rot
Fistulina hepatica v1.0	Fishe1	Agaricales	Brown-rot
Digitatispora marina 008cD1.1 v1.0	Digmar1	Atheliales	White-rot
Nia vibrissa CBS 119815 v1.0	Niavib1	Agaricales	Saprotroph
Flagelloscypha sp. PMI_526 v1.0	FlaPMI526_1	Agaricales	Ectomycorrhizal
Dendrothele microspora FP 101998 v1.0	Denmi1	Agaricales	White-rot
Pleurotus ostreatus PC15 v2.0	PleosPC15_2	Agaricales	White-rot
Pleurotus ostreatus PC9 v1.0	PleosPC9_1	Agaricales	White-rot
Pleurotus eryngii ATCC 90797 v1.0	Pleery1	Agaricales	White-rot
Aphanobasidium pseudotsugae OMC1630 v1.0	Aphpse1	Agaricales	White-rot
Radulomyces confluens OMC1631 v1.0	Radcon1	Agaricales	White-rot
Pterula gracilis CBS309.79 v1.0	Ptegra1	Agaricales	Saprotroph
Typhula sp. TRa3160C v1.0	TyphTRa3160C_1	Agaricales	Saprotroph
Anomoporia bombycina ATCC 64506 v1.0	Anobom1	Amylocorticiales	Brown-rot
Anomoporia kamtschatica OMC1758 v1.0	Anokam1	Amylocorticiales	Brown-rot
Amylocorticium subincarnatum OMC1654 v1.0	Amysub1	Amylocorticiales	Brown-rot
Plicaturopsis crispa v1.0	Plicr1	Amylocorticiales	White-rot
Intextomyces contiguus OMC1699 v1.0	Intcon1_1	Unclassified	White-rot
Serpulomyces borealis OMC1750 v1.0	Serbor1	Amylocorticiales	Brown-rot

Anomoloma albolutescens OMC1655 v1.0	Anoalb1	Amylocorticiales	White-rot
Anomoporia myceliosa MJL 4413 v1.0	Anomyc1	Amylocorticiales	Brown-rot
Piloderma byssinum 4S31 v1.0	Pilbys1	Atheliales	Ectomycorrhizal
Piloderma olivaceum F 1598 v1.0	Pilcr1	Atheliales	Ectomycorrhizal
Piloderma sphaerosporum 354 v1.0	Pilsph1	Atheliales	Ectomycorrhizal
Fibulorhizoctonia psychrophila CBS 109695 v1.0	Fibsp1	Atheliales	White-rot
Suillus tomentosus FC115 v1.0	Suitom1	Boletales	Ectomycorrhizal
Suillus hirtellus EM16 v1.0	Suihi1	Boletales	Ectomycorrhizal
Suillus variegatus UH-Sva-Z1 v1.0	Suivar1	Boletales	Ectomycorrhizal
Suillus fuscotomentosus FC203 v1.0	Suifus1	Boletales	Ectomycorrhizal
Suillus punctipes FC325 v1.0	Suipunc1	Boletales	Ectomycorrhizal
Suillus cf. *variegatus* FC465 v1.0	Suivari1	Boletales	Ectomycorrhizal
Suillus plorans S12 v1.0	Suiplo1	Boletales	Ectomycorrhizal
Suillus discolor FC423 v1.0	Suidis1	Boletales	Ectomycorrhizal
Suillus bovinus UH-Sbo-P2 v1.0	Suibov1	Boletales	Ectomycorrhizal
Suillus subalutaceus FC151 v1.0	Suisu1	Boletales	Ectomycorrhizal
Suillus cf. *subluteus* FC464 v1.0	Suisubl1	Boletales	Ectomycorrhizal
Suillus americanus EM31 v1.0	Suiame1	Boletales	Ectomycorrhizal
Suillus cf. *sibiricus* RUI384 v1.0	Suisib1	Boletales	Ectomycorrhizal
Suillus subaureus MN1 v1.0	Suisub1	Boletales	Ectomycorrhizal
Suillus cothurnatus VC 1858 v1.0	Suicot1	Boletales	Ectomycorrhizal
Suillus decipiens EM49 v1.0	Suidec1	Boletales	Ectomycorrhizal
Suillus spraguei EM44 v1.0	Suipic1	Boletales	Ectomycorrhizal
Suillus sp. nov. FC429 v1.0	SuiFC429_1	Boletales	Ectomycorrhizal
Suillus occidentalis FC124 v1.0	Suiocc1	Boletales	Ectomycorrhizal
Suillus pseudogranulatus FC45 v1.0	Suipse1	Boletales	Ectomycorrhizal
Suillus luteus UH-Slu-Lm8-n1 v3.0	Suilu4	Boletales	Ectomycorrhizal
Suillus brevipes Sb2 v2.0	Suibr2	Boletales	Ectomycorrhizal
Suillus kaibabensis FC96 v2.0	Suikai2	Boletales	Ectomycorrhizal
Suillus pungens FC27 v1.0	Suipun1	Boletales	Ectomycorrhizal
Suillus quiescens FC197 v1.0	Suiqui1	Boletales	Ectomycorrhizal
Suillus collinitus S28 v1.0	Suicol1	Boletales	Ectomycorrhizal
Suillus placidus DOB743 v1.0	Suipla1	Boletales	Ectomycorrhizal
Suillus placidus S07 v1.0	Suiplac1	Boletales	Ectomycorrhizal
Suillus weaverae EM37 v1.0	Suigr1	Boletales	Ectomycorrhizal
Suillus lakei FC43 v1.0	Suilak1	Boletales	Ectomycorrhizal
Suillus elbensis FC64 v1.0	Suielb1	Boletales	Ectomycorrhizal
Suillus viscidus S02 v2.0	Suivis2	Boletales	Ectomycorrhizal
Suillus clintonianus FC179 v1.0	Suicli1	Boletales	Ectomycorrhizal
Suillus ochraceoroseus FC498 v1.0	Suioch1	Boletales	Ectomycorrhizal
Suillus paluster FC165 v1.0	Suipal1	Boletales	Ectomycorrhizal
Suillus ampliporus FC55 v1.0	Suiamp1	Boletales	Ectomycorrhizal
Rhizopogon vesiculosus Smith	Rhives1	Boletales	Ectomycorrhizal
Rhizopogon vinicolor AM-OR11-026 v1.0	Rhivi1	Boletales	Ectomycorrhizal
Rhizopogon vulgaris FC72 v1.0	Rhivul1	Boletales	Ectomycorrhizal
Rhizopogon salebrosus TDB-379 v1.0	Rhisa1	Boletales	Ectomycorrhizal

Rhizopogon truncatus FC74 v1.0	Rhitru1	Boletales	Ectomycorrhizal
Pisolithus thermaeus 11 v1.0	Pisthe1	Boletales	Ectomycorrhizal
Pisolithus croceorrhizus subspA 74A v1.0	Piscro1	Boletales	Ectomycorrhizal
Pisolithus croceorrhizus ssp. 2 72 v1.0	Pisthe227_1	Boletales	Ectomycorrhizal
Pisolithus sp. B1 v1.0	Pismi_AB1_1	Boletales	Ectomycorrhizal
Pisolithus microcarpus 441 v1.0	Pismi1	Boletales	Ectomycorrhizal
Pisolithus microcarpus 441 v2.0	Pismi2	Boletales	Ectomycorrhizal
Pisolithus albus SI12 v1.0	Pisalb1	Boletales	Ectomycorrhizal
Pisolithus marmoratus ssp. 2 16A v1.0	Pismar1	Boletales	Ectomycorrhizal
Pisolithus tinctorius Marx 270 v1.0	Pisti1	Boletales	Ectomycorrhizal
Pisolithus tinctorius Marx 270 v2.0	Pisti2	Boletales	Ectomycorrhizal
Pisolithus orientalis OTSU v2.0	Pisori2	Boletales	Ectomycorrhizal
Scleroderma citrinum Foug A v1.0	Sclci1	Boletales	Ectomycorrhizal
Scleroderma citrinum hr. v1.0	Sclcihr1	Boletales	Ectomycorrhizal
Boletus edulis BED1 v4.0	Boled5	Boletales	Ectomycorrhizal
Boletus edulis Prilba v1.0	Boledp1	Boletales	Ectomycorrhizal
Xerocomus badius 84.06 v1.0	Xerba1	Boletales	Ectomycorrhizal
Boletus coccyginus 2016PMI039 v1.0	Bolcoc1	Boletales	Ectomycorrhizal
Chalciporus piperatus 201810 CP v1.0	Chapip1	Boletales	Mycoparasite
Paxillus ammoniavirescens Pou09.2 v1.0	Paxam1	Boletales	Ectomycorrhizal
Paxillus involutus ATCC 200175 v1.0	Paxin1	Boletales	Ectomycorrhizal
Paxillus adelphus Ve08.2h10 v2.0	Paxru2	Boletales	Ectomycorrhizal
Gyrodon lividus BX v1.0	Gyrli1	Boletales	Ectomycorrhizal
Melanogaster broomeianus MBLB.BST v1.0	Melbro1	Boletales	Ectomycorrhizal
Hydnomerulius pinastri v2.0	Hydpi2	Boletales	Brown-rot
Phlebopus sp. FC_14 v2.0	PhlFC14_2	Boletales	Brown-rot
Hygrophoropsis aurantiaca ATCC 28755 v1.0	Hygaur1	Boletales	Brown-rot
Leucogyrophana mollusca KUC20120723A-06 v1.0	Leumo1	Boletales	Brown-rot
Coniophora olivacea MUCL 20566 v1.0	Conol1	Boletales	Brown-rot
Coniophora puteana v1.0	Conpu1	Boletales	Brown-rot
Serpula lacrymans S7.3 v2.0	SerlaS7_3_2	Boletales	Brown-rot
Serpula lacrymans S7.9 v2.0	SerlaS7_9_2	Boletales	Brown-rot
Serpula himantioides (*S.lacrymans* var *shastensis*) MUCL38935 v1.0	Serla_varsha1	Boletales	Brown-rot
Pycnoporus coccineus CIRM-BRFM 310 v1.0	Pycco1	Polyporales	White-rot
Pycnoporus coccineus CIRM-BRFM 1662 v1.0	Pycco1662_1	Polyporales	White-rot
Pycnoporus sanguineus CIRM-BRFM 1264 v1.0	Pycsa1	Polyporales	White-rot
Pycnoporus cinnabarinus CIRM-BRFM 137 v1.0	Pycci1	Polyporales	White-rot
Pycnoporus cinnabarinus CIRM-BRFM 50 v1.0	Pyccin1	Polyporales	White-rot
Pycnoporus puniceus CIRM-BRFM 1868 v1.0	Pycpun1	Polyporales	White-rot
Leiotrametes sp. CIRM-BRFM 1775 v1.0	Leisp1	Polyporales	White-rot
Leiotrametes menziesii CIRM-BRFM 1781 v1.0	Tramen1	Polyporales	White-rot
Leiotrametes lactinea CIRM-BRFM 1664 v1.0	Tralac1	Polyporales	White-rot
Trametes cingulata CIRM-BRFM 1805 v1.0	Traci1	Polyporales	White-rot
Trametes ljubarskyi CIRM-BRFM 1659 v1.0	Tralj1	Polyporales	White-rot
Trametes pubescens FBCC735	Trapub1	Polyporales	White-rot
Trametes versicolor v1.0	Trave1	Polyporales	White-rot

Trametes betulina CIRM-BRFM 1801 v1.0	Trabet1	Polyporales	White-rot
Trametes gibbosa CIRM-BRFM 1770 v1.0	Tragib1	Polyporales	White-rot
Trametes polyzona CIRM-BRFM 1798 v1.0	Trapol1	Polyporales	White-rot
Trametes maxima CIRM-BRFM 1813 v1.0	Tramax1	Polyporales	White-rot
Trametes meyenii CIRM-BRFM 1810 v1.0	Tramey1	Polyporales	White-rot
Artolenzites elegans CIRM-BRFM 1663 v1.0	Artel1	Polyporales	White-rot
Artolenzites elegans CIRM-BRFM 1122 v1.0	Artele1122_1	Polyporales	White-rot
Lentinus tigrinus ALCF2SS1-6 v1.0	Lenti6_1	Polyporales	White-rot
Lentinus tigrinus ALCF2SS1-7 v1.0	Lenti7_1	Polyporales	White-rot
Lentinus tigrinus v1.0	Sisbr1	Polyporales	White-rot
Polyporus arcularius v1.0	Polar1	Polyporales	White-rot
Polyporus brumalis CIRM-BRFM 1820 v1.0	Polbr1	Polyporales	White-rot
Polyporus squamosus CCBS 676 v1.0	Polsqu1	Polyporales	White-rot
Earliella scabrosa CIRM-BRFM 1817 v1.0	Earsca1	Polyporales	White-rot
Fomes fomentarius CIRM-BRFM 1821 v1.0	Fomfom1	Polyporales	White-rot
Hexagonia nitida CIRM-BRFM 1802 v1.0	Hexnit1	Polyporales	White-rot
Dichomitus squalens CBS463.89 v1.0	Dicsqu463_1	Polyporales	White-rot
Dichomitus squalens CBS464.89 v1.0	Dicsqu464_1	Polyporales	White-rot
Dichomitus squalens LYAD-421 SS1 v1.0	Dicsq1	Polyporales	White-rot
Dichomitus squalens OM18370.1 v1.0	Dicsqu18370_1	Polyporales	White-rot
Perenniporia subacida PersubVin-SM18 v1.0	Persub1	Polyporales	White-rot
Fomitopsis rosea Sig1Fomros20-ss v1.0	Fomro1	Polyporales	Brown-rot
Rhodofomes roseus CIRM-BRFM 1785 v1.0	Fomros1	Polyporales	Brown-rot
Antrodia serialis Sig1Antser10 v1.0	Antser1	Polyporales	Brown-rot
Fomitopsis pinicola FP-58527 SS1 v3.0	Fompi3	Polyporales	Brown-rot
Fomitopsis betulina CIRM-BRFM 1772 v1.1	Pipbet1_1	Polyporales	Brown-rot
Daedalea quercina v1.0	Daequ1	Polyporales	Brown-rot
Postia placenta MAD 698-R v1.0	Pospl1	Polyporales	Brown-rot
Postia placenta MAD-698-R-SB12 v1.0	PosplRSB12_1	Polyporales	Brown-rot
Antrodia sinuosa LB1 v1.0	Antsi1	Polyporales	Brown-rot
Fibroporia radiculosa TFFH 294	Fibra1	Polyporales	Brown-rot
Laetiporus sulphureus var. *sulphureus* v1.0	Laesu1	Polyporales	Brown-rot
Wolfiporia cocos MD-104 SS10 v1.0	Wolco1	Polyporales	Brown-rot
Pycnoporellus fulgens PcyfulGre-SM17 v1.0	Pycful1	Polyporales	Brown-rot
Sparassis latifolia CCMJ1100 v1.0	Spalat1	Polyporales	Brown-rot
Amylocystis lapponica SKaAmylap13 v1.0	Amylap1_1	Polyporales	Brown-rot
Postia stiptica OMC1664 v1.0	Posst1	Polyporales	Brown-rot
Ceriporiopsis (*Gelatoporia*) *subvermispora* B	Cersu1	Polyporales	White-rot
Obba rivulosa 3A-2 v1.0	Obbri1	Polyporales	White-rot
Flavodon flavus 38 v1.0	Flafl1	Polyporales	White-rot
Irpex lacteus CCBAS Fr. 238 617/93 v1.0	Irplac1	Polyporales	White-rot
Hydnopolyporus fimbriatus CBS384.51 v1.0	Hydfim1	Polyporales	White-rot
Cytidiella melzeri FP 102339 v1.0	Cytmel1	Polyporales	White-rot
Trametopsis cervina CIRM-BRFM 1824 v1.0	Trace1	Polyporales	White-rot
Ceriporia viridans OMC1683 v1.0	Cervir1	Polyporales	White-rot
Leptoporus mollis OMC1684 v1.0	Lepmol1	Polyporales	Brown-rot

Phanerochaete chrysosporium RP-78 v2.2	Phchr2	Polyporales	White-rot
Phanerochaete chrysosporium RP-78 v3.0	Phchr3_2	Polyporales	White-rot
Phanerochaete carnosa HHB-10118-Sp v1.0	Phaca1	Polyporales	White-rot
Phlebiopsis gigantea v1.0	Phlgi1	Polyporales	White-rot
Bjerkandera adusta v1.0	Bjead1_1	Polyporales	White-rot
Phlebia radiata Fr. (isolate 79, FBCC0043)	Phlrad1	Polyporales	White-rot
Phlebia centrifuga FBCC195	Phlcen1	Polyporales	White-rot
Abortiporus biennis CCBS 521 v1.0	Abobi1	Polyporales	White-rot
Abortiporus biennis CIRM-BRFM 1778 v1.0	Abobie1	Polyporales	White-rot
Meripilus sumstinei FP 105329 v1.0	Mersum1	Polyporales	White-rot
Climacocystis borealis CliBor001 v1.0	Clibor1	Polyporales	White-rot
Cerrena unicolor v1.1	Cerun2	Polyporales	White-rot
Panus rudis PR-1116 ss-1 v1.0	Panru1	Polyporales	White-rot
Antrodiella citrinella FBCC 969 v1.0	Antcit1	Polyporales	White-rot
Epithele typhae CBS 203.58 v1.0	Epityp1	Polyporales	White-rot
Crustoderma dryinum OMC1663 v1.0	Crudry1	Polyporales	Brown-rot
Albatrellus peckianus DAOM 52321 v1.0	Albpec1	Russulales	Ectomycorrhizal
Cristinia sonorae KKN 215 v1.0	Crison1	Atheliales	White-rot
Thelephora ganbajun P2 v1.0	Thega1	Thelephorales	Ectomycorrhizal
Thelephora terrestris UH-Tt-Lm1 v1.0	Theter1	Thelephorales	Ectomycorrhizal
Russula emetica Prilba v1.0	Ruseme1	Russulales	Ectomycorrhizal
Russula rugulosa BPL654 v1.0	Rusrug1	Russulales	Ectomycorrhizal
Russula seminuda PSC4341-TAS1 v1.0	Russem1	Russulales	Ectomycorrhizal
Russula ochroleuca Prilba v1.0	Rusoch1	Russulales	Ectomycorrhizal
Russula vinacea BPL710 v1.0	Rusvin1	Russulales	Ectomycorrhizal
Russula dissimulans BPL704 v1.0	Rusdis1	Russulales	Ectomycorrhizal
Russula earlei BPL698 v1.0	Rusear1	Russulales	Ectomycorrhizal
Russula brevipes BPL707 v1.0	Rusbre1	Russulales	Ectomycorrhizal
Russula compacta BPL669 v1.0	Ruscom1	Russulales	Ectomycorrhizal
Lactifluus cf. *subvellereus* BPL653 v1.0	Lacsub1	Russulales	Ectomycorrhizal
Lactifluus cf. *volemus* BPL652 v1.0	Lacvol1	Russulales	Ectomycorrhizal
Multifurca ochricompacta BPL690 v1.0	Muloch1	Russulales	Ectomycorrhizal
Lactarius hengduanensis 84 v1.0	Lachen1	Russulales	Ectomycorrhizal
Lactarius pseudohatsudake 88 v1.0	Lacpse1	Russulales	Ectomycorrhizal
Lactarius akahatsu QP v1.0	Lacaka1	Russulales	Ectomycorrhizal
Lactarius deliciosus 48 v1.0	Lacdel1	Russulales	Ectomycorrhizal
Lactarius hatsudake 109 v1.0	Lachat1	Russulales	Ectomycorrhizal
Lactarius sanguifluus B21 v1.0	Lacsan1	Russulales	Ectomycorrhizal
Lactarius vividus 141 v1.0	Lacviv1	Russulales	Ectomycorrhizal
Lactarius indigo 2018DUKE089 v1.0	Lacind1	Russulales	Ectomycorrhizal
Lactarius psammicola BPL869 v1.0	Lacpsa1	Russulales	Ectomycorrhizal
Lactarius quietus S23C v1.0	Lacqui1	Russulales	Ectomycorrhizal
Gloeopeniophorella convolvens OM19405 v1.0	Glocon1	Russulales	White-rot
Auriscalpium vulgare FP105234-Sp v1.0	Aurvu1	Russulales	White-rot
Lentinellus vulpinus AHS73672-sp v1.0	Lenvul1	Russulales	White-rot
Clavicorona pyxidata HHB10654 v1.0	Clapy1	Russulales	White-rot

Peniophora sp. CONTA v1.0	Lopni1	Russulales	White-rot
Peniophora sp. v1.0	Ricme1	Russulales	White-rot
Scytinostroma sp. KUC9335 v1.0	Scysp1_1	Russulales	White-rot
Vararia minispora EC-137 v1.0	Varmin1	Russulales	White-rot
Amylostereum chailletii DWAch2 v1.0	Amycha1	Russulales	White-rot
Echinodontium tinctorium Aho-80 v1.0	Echtin1	Russulales	White-rot
Dentipellis sp. KUC8613 v1.0	Densp1	Russulales	White-rot
Laxitextum bicolor CBS 258.73 v1.0	Laxbic1	Russulales	White-rot
Hericium coralloides FP-101451 v1.0	Hercor1	Russulales	White-rot
Heterobasidion annosum v2.0	Hetan2	Russulales	White-rot
Stereum hirsutum FP-91666 SS1 v1.0	Stehi1	Russulales	White-rot
Heliocybe sulcata OMC1185 v1.0	Helsul1	Gloeophyllales	Brown-rot
Neolentinus lepideus v1.0	Neole1	Gloeophyllales	Brown-rot
Gloeophyllum trabeum v1.0	Glotr1_1	Gloeophyllales	Brown-rot
Veluticeps abietina OMC1657 v1.0	Velabi1	Gloeophyllales	Brown-rot
Jaapia argillacea v1.0	Jaaar1	Jaapiales	Brown-rot
Boreostereum radiatum TFC1981-005 v1.0	Borrad1	Gloeophyllales	Brown-rot
Vuilleminia comedens VcCUCC2015_SSI3 v1.0	Vuico1	Corticiales	White-rot
Vuilleminia comedens VcCUCC2015_SSI6 v1.0	Vuicom1	Corticiales	White-rot
Punctularia strigosozonata v1.0	Punst1	Corticiales	White-rot
Galzinia incrustans CBS 104901 v1.0	Galinc1	Corticiales	White-rot
Xenasmatella tulasnelloidea OMC1662 v1.0	Xentul1	Corticiales	White-rot
Xenasmatella vaga CBS212.54 v1.0	Xenvag1	Unclassified	White-rot
Phlebia subcretacea OMC74 v1.0	Phlsub1	Polyporales	White-rot
Porodaedalea chrysoloma FP-135951 v1.0	Porchr1	Hymenochaetales	White-rot
Porodaedalea niemelaei PN71-100-IP13 v1.0	Pornie1_2	Hymenochaetales	White-rot
Phellinus igniarius CCBS 575 v1.0	Pheign1_1	Hymenochaetales	White-rot
Fomitiporia mediterranea v1.0	Fomme1	Hymenochaetales	White-rot
Onnia scaura P-53A v1.0	Onnsc1	Hymenochaetales	White-rot
Phellopilus nigrolimitatus SigPhenig9 v1.0	Pheni1	Hymenochaetales	White-rot
Phellinus ferrugineofuscus SpK3Phefer14 v1.0	Phefer1	Hymenochaetales	White-rot
Phellinus viticola PhevitSig-SM15 v1.0	Phevit1	Hymenochaetales	Saprotroph
Trichaptum abietinum v1.0	Triab1_1	Hymenochaetales	White-rot
Schizopora paradoxa KUC8140 v1.0	Schpa1	Hymenochaetales	White-rot
Rigidoporus microporus ED310 v1.0	Rigmic1	Polyporales	White-rot
Rickenella fibula HBK330-10 v1.0	Ricfib1	Hymenochaetales	Plant-pathogen
Rickenella mellea v1.0 (SZMC22713)	Ricmel1	Hymenochaetales	Plant-pathogen
Kurtia argillacea OMC1749 v1.0	Kurarg1	Corticiales	Ectomycorrhizal
Resinicium bicolor OMC78 v1.0	Resbic1	Hymenochaetales	White-rot
Sidera vulgaris OMC 1730 v1.0	Sidvul1	Hymenochaetales	White-rot
Sistotremastrum niveocremeum HHB9708 ss-1 1.0	Sisni1	Trechisporales	White-rot
Sistotremastrum suecicum v1.0	Sissu1	Trechisporales	White-rot
Porpomyces mucidus OMC1666 v1.0	Pormuc1	Trechisporales	White-rot
Geastrum triplex GST.BST v1.0	Geatri1	Geastrales	Saprotroph
Sclerogaster hysterangioides SCL2.BST v1.0	Sclhys1_1	Unclassified	Ectomycorrhizal
Sphaerobolus stellatus v1.0	Sphst1	Geastrales	White-rot

Gautieria morchelliformis GMNE.BST v1.0	Gaumor1_1	Gomphales	Ectomycorrhizal
Ramaria rubella (*R. acris*) UT-36052-T v1.0	Ramac1	Gomphales	Ectomycorrhizal
Hysterangium stoloniferum HS.BST v1.0	Hyssto1	Hysterangiales	Ectomycorrhizal
Mutinus elegans ME.BST v1.0	Mutel1	Phallales	Litter-decomposing
Aporpium caryae L-13461	Elmca1	Auriculariales	Saprotroph
Exidia glandulosa v1.0	Exigl1	Auriculariales	White-rot
Auricularia subglabra v2.0	Aurde3_1	Auriculariales	White-rot
Oliveonia pauxilla MPI-PUGE-AT-0066 v1.0	Olipa1	Auriculariales	Saprotroph
Piriformospora indica DSM 11827 from MPI	Pirin1	Sebacinales	Ectomycorrhizal
Serendipita sp. 407 v1.0	Serend1	Sebacinales	Ectomycorrhizal
Sebacina vermifera MAFF 305830 v1.0	Sebve1	Sebacinales	Ectomycorrhizal
Serendipita vermifera ssp. *bescii* NFPB0129 v1.0	Sebvebe1	Sebacinales	Ectomycorrhizal
Ceratobasidium sp. 423 v1.0	Cersp423_1	Cantharellales	Ectomycorrhizal
Thanatephorus cucumeris MPI-SDFR-AT-0096 v1.0	Thacu1	Cantharellales	Plant-pathogen
Rhizoctonia solani AG-1 IB	Rhiso1	Cantharellales	Plant-pathogen
Ceratobasidium sp. 379 v1.0	Cersp379_1	Cantharellales	Ectomycorrhizal
Ceratobasidium sp. 394 v1.0	Cersp394_1	Cantharellales	Ectomycorrhizal
Ceratobasidium sp. anastomosis group I; DN8442 v1.0	CerAGI	Cantharellales	Ectomycorrhizal
Tulasnella calospora AL13/4D v1.0	Tulca1	Cantharellales	Ectomycorrhizal
Tulasnella calospora UAMH 9824 v1.0	Tulcal1	Cantharellales	Ectomycorrhizal
Tulasnella sp. 425 v1.0	Tulsp425_1	Cantharellales	Ectomycorrhizal
Tulasnella inquilina UAMH 7632 v1.0	Tulinq1	Cantharellales	Ectomycorrhizal
Hydnum rufescens UP504 v2.0	Hydru2	Cantharellales	Ectomycorrhizal
Sistotrema muscicola OMC 1658 v1.0	Sismus1	Corticiales	Ectomycorrhizal
Sistotrema raduloides OMC1660 v1.0	Sisrad1	Corticiales	White-rot
Sistotrema sp. PMI_390 v1.0	ClaPMI390	Corticiales	White-rot
Cantharellales sp. MUCL 035158 v1.0	Tylas1	Cantharellales	Unknown
Cantharellus anzutake C23 v1.0	Cananz1	Cantharellales	Ectomycorrhizal
Sistotrema sernanderi OMC 1753 v1.0	Sisser1	Corticiales	White-rot
Botryobasidium botryosum v1.0	Botbo1	Cantharellales	White-rot
Calocera cornea v1.0	Calco1	Dacrymycetales	Brown-rot
Calocera viscosa v1.0	Calvi1	Dacrymycetales	Brown-rot
Cerinomyces ceraceus ATCC 56525 v1.0	Cercer1	Dacrymycetales	Brown-rot
Cerinomyces crustulinus OMC1686 v1.0	Cercru1	Dacrymycetales	Brown-rot
Dacryonaema rufum OMC1653 v1.0	Dacruf1	Dacrymycetales	Brown-rot
Dacryopinax primogenitus DJM 731 SSP1 v1.0	Dacsp1	Dacrymycetales	Brown-rot
Dacrymyces fennicus OMC1656 v1.0	Dactor1	Dacrymycetales	Brown-rot
Unilacryma unispora MAFF 240146 v1.0	Uniuni1	Dacrymycetales	Brown-rot
Dacrymyces tortus OMC1693 v1.0	Dactort1	Dacrymycetales	Brown-rot
Ganoderma leucocontextum Dai12418 v1.0	Ganleu1	Polyporales	White-rot
Lentinula aciculospora JLM2183 v1.0	LacJLM2183_1	Agaricales	White-rot
Lentinula edodes B17 v1.1	Lened_B_1_1	Agaricales	White-rot
Phlebopus sp. FC_14 v1.0	Pis20_1	Boletales	Brown-rot

Table S2 Gini importance of all CAZymes in RF models predicting decay modes.

CAZymes	Original		Over-sampled	
	mean	var	mean	var
AA9	0.998	0.000	0.995	0.000
GH7	0.703	0.015	0.783	0.013
AA2	0.505	0.009	0.730	0.013
GH6	0.590	0.007	0.553	0.006
AA5_1	0.264	0.003	0.536	0.009
CBM1	0.580	0.006	0.536	0.006
AA5	0.230	0.003	0.444	0.007
CE1	0.306	0.007	0.444	0.010
GH10	0.216	0.003	0.418	0.006
PL4	0.190	0.002	0.397	0.008
AA8	0.562	0.007	0.390	0.006
CE15	0.247	0.005	0.332	0.005
GH74	0.342	0.004	0.299	0.004
GH131	0.283	0.003	0.282	0.003
AA3_1	0.371	0.004	0.255	0.003
GH5_9	0.143	0.002	0.247	0.004
CE12	0.115	0.001	0.232	0.003
AA14	0.089	0.001	0.232	0.004
GH35	0.095	0.001	0.173	0.003
PL1	0.080	0.001	0.167	0.002
GH79	0.104	0.001	0.159	0.003
GH15	0.094	0.001	0.156	0.003
GT1	0.120	0.002	0.139	0.002
GH16	0.111	0.001	0.134	0.002
GH135	0.041	0.000	0.133	0.002
AA3	0.175	0.003	0.123	0.002
AA3_2	0.137	0.002	0.116	0.001
GH45	0.077	0.001	0.114	0.002
CE8	0.039	0.000	0.111	0.002
AA1_1	0.067	0.001	0.110	0.001
GH18	0.104	0.002	0.107	0.002
GH145	0.076	0.001	0.085	0.001
GH11	0.044	0.000	0.083	0.001
CBM13	0.090	0.001	0.078	0.001
GH92	0.067	0.001	0.078	0.001
GT15	0.041	0.000	0.078	0.001
PL1_7	0.032	0.000	0.073	0.001
GT8	0.050	0.000	0.070	0.000
GH17	0.041	0.000	0.070	0.001
CBM20	0.059	0.000	0.069	0.001
AA1	0.058	0.001	0.067	0.001
GH43	0.066	0.000	0.067	0.000
PL8_4	0.032	0.000	0.055	0.000
GH93	0.023	0.000	0.053	0.000
PL14_4	0.052	0.000	0.052	0.000
PL4_1	0.027	0.000	0.051	0.000
GH44	0.025	0.000	0.049	0.000
GT17	0.028	0.000	0.048	0.000
PL4_3	0.011	0.000	0.045	0.000
PL8	0.032	0.000	0.044	0.000
GH25	0.058	0.000	0.043	0.000
EXPN	0.054	0.000	0.043	0.000
GH152	0.033	0.000	0.042	0.000
CE16	0.049	0.000	0.041	0.000
GH12	0.036	0.000	0.040	0.000
GH13_32	0.117	0.002	0.039	0.000
PL3	0.019	0.000	0.038	0.000
PL14	0.037	0.000	0.037	0.000
AA3_4	0.012	0.000	0.035	0.000
GH128	0.046	0.000	0.034	0.000
PL3_2	0.024	0.000	0.034	0.000
AA4	0.093	0.002	0.034	0.000
GT22	0.042	0.000	0.034	0.000
CE9	0.054	0.001	0.033	0.000
GH55	0.058	0.000	0.033	0.000
GH71	0.035	0.000	0.032	0.000
GH105	0.020	0.000	0.032	0.000
GH47	0.032	0.000	0.031	0.000
GH13_1	0.028	0.000	0.030	0.000
GH30	0.026	0.000	0.029	0.000
GH20	0.017	0.000	0.028	0.000
GH28	0.034	0.000	0.028	0.000
GH27	0.035	0.000	0.026	0.000
GH5	0.034	0.000	0.026	0.000
CBM5	0.050	0.000	0.026	0.000
GH13	0.027	0.000	0.023	0.000
AA3_3	0.031	0.000	0.022	0.000
GH51	0.019	0.000	0.021	0.000
GH5_5	0.031	0.000	0.021	0.000
GH76	0.025	0.000	0.021	0.000
AA12	0.015	0.000	0.021	0.000
GH2	0.026	0.000	0.021	0.000
GH89	0.058	0.001	0.020	0.000
CBM35	0.039	0.000	0.019	0.000
GT90	0.024	0.000	0.019	0.000
CBM50	0.036	0.000	0.019	0.000
AA16	0.041	0.000	0.019	0.000
GH3	0.029	0.000	0.019	0.000
AA6	0.014	0.000	0.018	0.000

CAZymes	Original		Over-sampled	
	mean	var	mean	var
GH95	0.013	0.000	0.018	0.000
GH30_dist	0.017	0.000	0.018	0.000
GH53	0.014	0.000	0.018	0.000
AA7	0.017	0.000	0.018	0.000
GH5_30	0.021	0.000	0.017	0.000
GH75	0.007	0.000	0.017	0.000
CE5	0.015	0.000	0.017	0.000
CBM67	0.007	0.000	0.017	0.000
GH30_3	0.018	0.000	0.016	0.000
CE4	0.026	0.000	0.016	0.000
GH31	0.018	0.000	0.016	0.000
AA1_2	0.032	0.000	0.016	0.000
GH37	0.019	0.000	0.015	0.000
GH13_40	0.014	0.000	0.015	0.000
GH78	0.025	0.000	0.015	0.000
GT48	0.025	0.000	0.015	0.000
GH29	0.020	0.000	0.014	0.000
GH1	0.020	0.000	0.014	0.000
CBM48	0.011	0.000	0.014	0.000
GH5_7	0.021	0.000	0.014	0.000
CBM21	0.016	0.000	0.014	0.000
AA7_dist	0.015	0.000	0.014	0.000
GT69	0.018	0.000	0.013	0.000
GH9	0.020	0.000	0.013	0.000
GT4	0.021	0.000	0.013	0.000
GT2	0.026	0.000	0.012	0.000
PL26	0.007	0.000	0.012	0.000
GH13_22	0.015	0.000	0.012	0.000
GH88	0.008	0.000	0.012	0.000
CBM18	0.011	0.000	0.012	0.000
GH5_50	0.017	0.000	0.012	0.000
myosin_motor	0.055	0.001	0.012	0.000
GH23	0.019	0.000	0.011	0.000
AA3_dist	0.025	0.000	0.010	0.000
GT41	0.007	0.000	0.009	0.000
GH5_DIST	0.041	0.000	0.009	0.000
GH65	0.024	0.000	0.009	0.000
GT32	0.012	0.000	0.009	0.000
GH81	0.012	0.000	0.009	0.000
GH5_31	0.021	0.000	0.009	0.000
PL35	0.009	0.000	0.009	0.000
GH115	0.013	0.000	0.009	0.000
GH125	0.005	0.000	0.007	0.000
GH5_15	0.014	0.000	0.007	0.000
GH5_12	0.014	0.000	0.007	0.000
GH5_22	0.006	0.000	0.007	0.000
GH26	0.026	0.000	0.007	0.000
CBM32	0.020	0.000	0.007	0.000
GH127	0.010	0.000	0.007	0.000
PL38	0.007	0.000	0.007	0.000
GH114	0.006	0.000	0.006	0.000
GT5	0.011	0.000	0.006	0.000
CBM12	0.005	0.000	0.006	0.000
GT20	0.013	0.000	0.006	0.000
GH36	0.017	0.000	0.006	0.000
GH13_5	0.007	0.000	0.005	0.000
GT76	0.009	0.000	0.005	0.000
GH62	0.005	0.000	0.004	0.000
GH30_7	0.003	0.000	0.004	0.000
PL1_2	0.003	0.000	0.004	0.000
GT31	0.007	0.000	0.004	0.000
GH32	0.006	0.000	0.004	0.000
GH133	0.013	0.000	0.004	0.000
PL9_3	0.004	0.000	0.004	0.000
AA11	0.003	0.000	0.004	0.000
GH72	0.012	0.000	0.004	0.000
CBM43	0.012	0.000	0.004	0.000
PL9	0.003	0.000	0.003	0.000
PL14_3	0.007	0.000	0.003	0.000
GH39	0.002	0.000	0.003	0.000
GT3	0.008	0.000	0.003	0.000
CE3	0.004	0.000	0.003	0.000
GT39	0.003	0.000	0.003	0.000
PL14_5	0.004	0.000	0.003	0.000
AA2_dist	0.005	0.000	0.003	0.000
GT71	0.006	0.000	0.003	0.000
GH38	0.002	0.000	0.003	0.000
GT35	0.003	0.000	0.003	0.000
GH30_5	0.001	0.000	0.002	0.000
GT49	0.014	0.000	0.002	0.000
GT50	0.006	0.000	0.002	0.000
PL7_4	0.001	0.000	0.002	0.000
GH5_11	0.002	0.000	0.002	0.000
GT66	0.005	0.000	0.002	0.000
PL7	0.002	0.000	0.002	0.000
GT47	0.005	0.000	0.002	0.000
GH85	0.003	0.000	0.002	0.000
GH24	0.001	0.000	0.002	0.000
CBM63	0.002	0.000	0.002	0.000

CAZymes	Original		Over-sampled	
	mean	var	mean	var
GH5_27	0.004	0.000	0.001	0.000
GT33	0.001	0.000	0.001	0.000
CBM38	0.006	0.000	0.001	0.000
GH140	0.001	0.000	0.001	0.000
GH154	0.004	0.000	0.001	0.000
GT24	0.002	0.000	0.001	0.000
PL1_4	0.001	0.000	0.001	0.000
GH106	0.002	0.000	0.001	0.000
AA1_dist	0.001	0.000	0.001	0.000
GH13_25	0.001	0.000	0.001	0.000
CBM19	0.001	0.000	0.001	0.000
GH54	0.001	0.000	0.001	0.000
CBM42	0.001	0.000	0.001	0.000
GH134	0.001	0.000	0.001	0.000
GH13_8	0.001	0.000	0.001	0.000
GH130	0.005	0.000	0.001	0.000
GT57	0.001	0.000	0.001	0.000
GT59	0.001	0.000	0.001	0.000
GT61	0.004	0.000	0.001	0.000
GH5_51	0.000	0.000	0.001	0.000
GH50	0.001	0.000	0.000	0.000
GT25	0.000	0.000	0.000	0.000
GH63	0.001	0.000	0.000	0.000
GT58	0.001	0.000	0.000	0.000
GH57	0.004	0.000	0.000	0.000
GH5_4	0.000	0.000	0.000	0.000
GH13_31	0.002	0.000	0.000	0.000
AA10	0.000	0.000	0.000	0.000
PL1_9	0.000	0.000	0.000	0.000
GT21	0.000	0.000	0.000	0.000
CBM52	0.000	0.000	0.000	0.000
PL20	0.000	0.000	0.000	0.000
GH162	0.000	0.000	0.000	0.000
GH46	0.000	0.000	0.000	0.000
GH94	0.000	0.000	0.000	0.000
GH67	0.000	0.000	0.000	0.000
AA9_dist	0.000	0.000	0.000	0.000
PL1_10	0.000	0.000	0.000	0.000
AA1_3	0.000	0.000	0.000	0.000
PL4_5	0.000	0.000	0.000	0.000
PL14_dist	0.000	0.000	0.000	0.000
GH42	0.000	0.000	0.000	0.000
GT23	0.000	0.000	0.000	0.000
CBM8	0.000	0.000	0.000	0.000
GH52	0.000	0.000	0.000	0.000
GH146	0.000	0.000	0.000	0.000
CBM66	0.000	0.000	0.000	0.000
CE2	0.000	0.000	0.000	0.000
AA4_dist	0.000	0.000	0.000	0.000
AA5_2	0.000	0.000	0.000	0.000
AA5_dist	0.000	0.000	0.000	0.000
AA8_dist	0.000	0.000	0.000	0.000
AA12_dist	0.000	0.000	0.000	0.000
AA13	0.000	0.000	0.000	0.000
CBM85	0.000	0.000	0.000	0.000
CBM6	0.000	0.000	0.000	0.000
CBM14	0.000	0.000	0.000	0.000
CBM16	0.000	0.000	0.000	0.000
CBM24	0.000	0.000	0.000	0.000
CBM57	0.000	0.000	0.000	0.000
CE6	0.000	0.000	0.000	0.000
CE11	0.000	0.000	0.000	0.000
CE14	0.000	0.000	0.000	0.000
GH5_39	0.000	0.000	0.000	0.000
GH87	0.000	0.000	0.000	0.000
GH158	0.000	0.000	0.000	0.000
GH5_16	0.000	0.000	0.000	0.000
GH5_46	0.000	0.000	0.000	0.000
GH13_m42	0.000	0.000	0.000	0.000
GH33	0.000	0.000	0.000	0.000
GH49	0.000	0.000	0.000	0.000
GH77	0.000	0.000	0.000	0.000
GH97	0.000	0.000	0.000	0.000
GH141	0.000	0.000	0.000	0.000
GH142	0.000	0.000	0.000	0.000
GT9	0.000	0.000	0.000	0.000
GT18	0.000	0.000	0.000	0.000
GT34	0.000	0.000	0.000	0.000
GT51	0.000	0.000	0.000	0.000
GT84	0.000	0.000	0.000	0.000
PL6_1	0.000	0.000	0.000	0.000
PL17_2	0.000	0.000	0.000	0.000
PL35_dist	0.000	0.000	0.000	0.000
PL1_6	0.000	0.000	0.000	0.000
PL1_dist	0.000	0.000	0.000	0.000
PL4_dist	0.000	0.000	0.000	0.000
PL9_1	0.000	0.000	0.000	0.000
PL38_dist	0.000	0.000	0.000	0.000

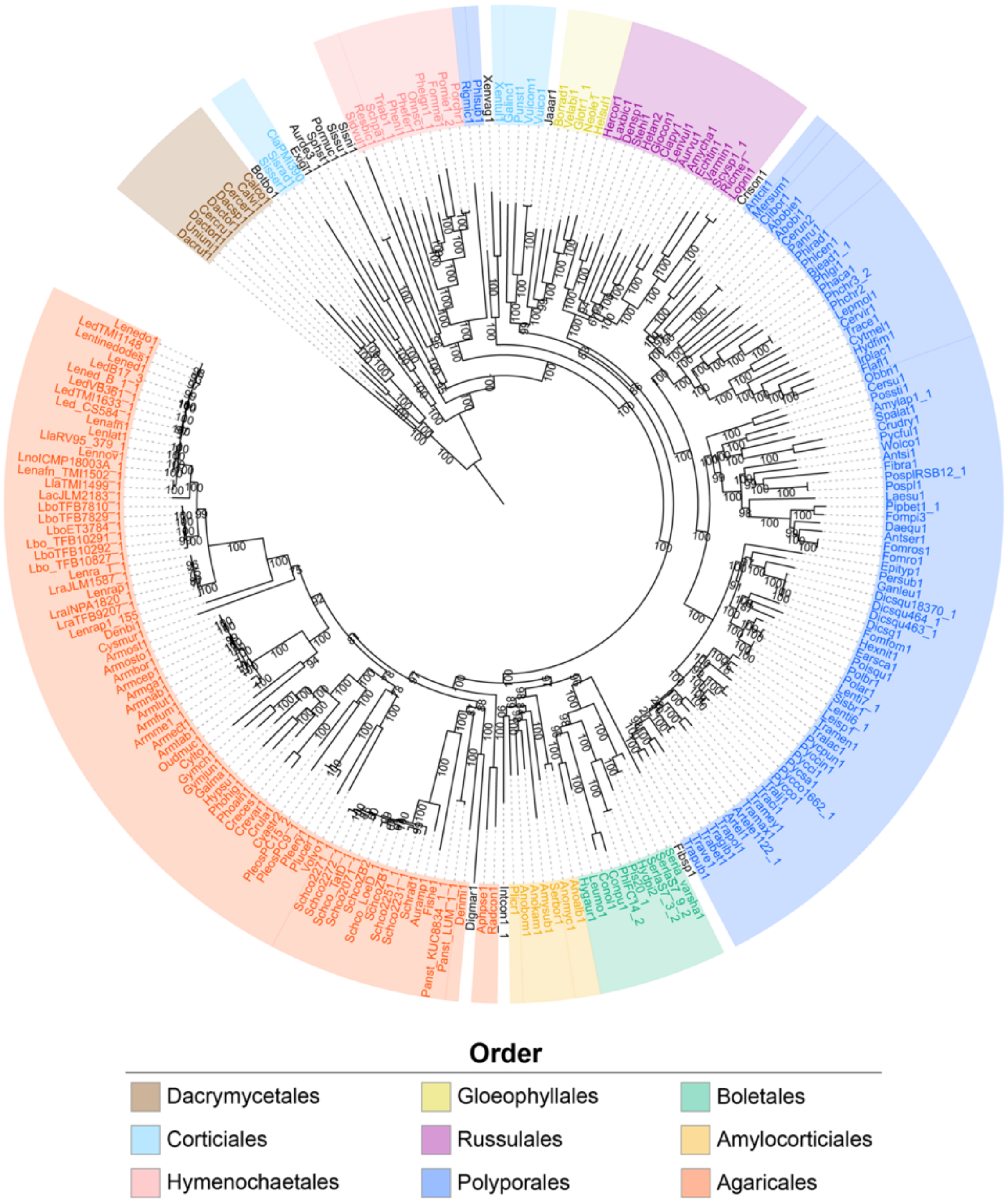

Figure S1 Phylogenetic tree of the wood-rotting fungi.

Bootstrap values for each branch in the phylogenetic tree estimation. Most of the branches for divisions down to the eye level had high confidence of 95% or more; the divisions of Polyporales and Russulales (86%) and the clade consisting of these two orders and a clade of Gloeophyllales and Corticiales (83%) had slightly lower values.

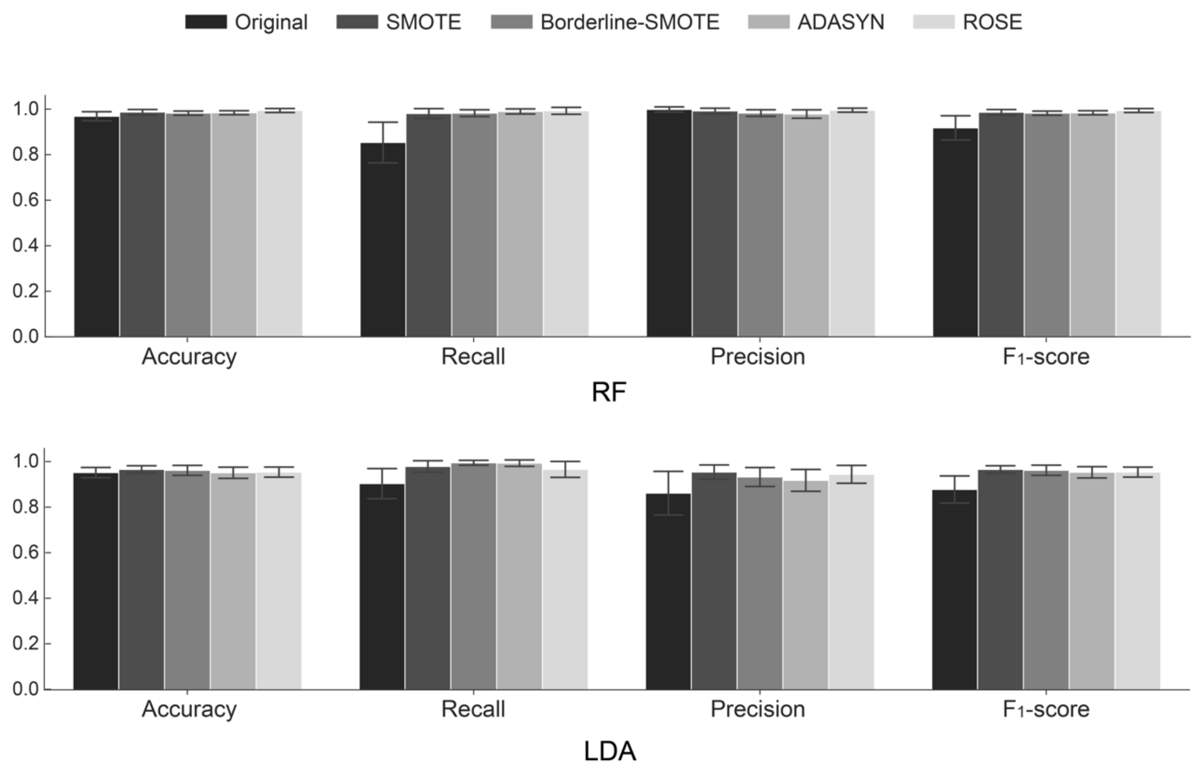

Figure S2 Choice of oversampling technique used in this study.

We oversampled the dataset using four methods (SMOTE1, Borderline SMOTE2, ADASYN3, and ROSE4) from the Python library imbalanced-learn and compared the performance of the models trained on them. The dataset was split 7:3 into training and test data, and the correctness, fit, recall, and F-measure of the LDA or RF models built from the training data were computed for the test data using LinearDiscriminantAnalysis and RandomForestClassifier from the Python library scikit-learn, respectively. The process was repeated a total of 100 times with randomized oversampling and data splitting.

Error bars are standard deviations.

All four oversampling methods were effective in eliminating bias in the model's predictive ability, but SMOTE, the most effective and orthodox oversampling method, was used in subsequent experiments.

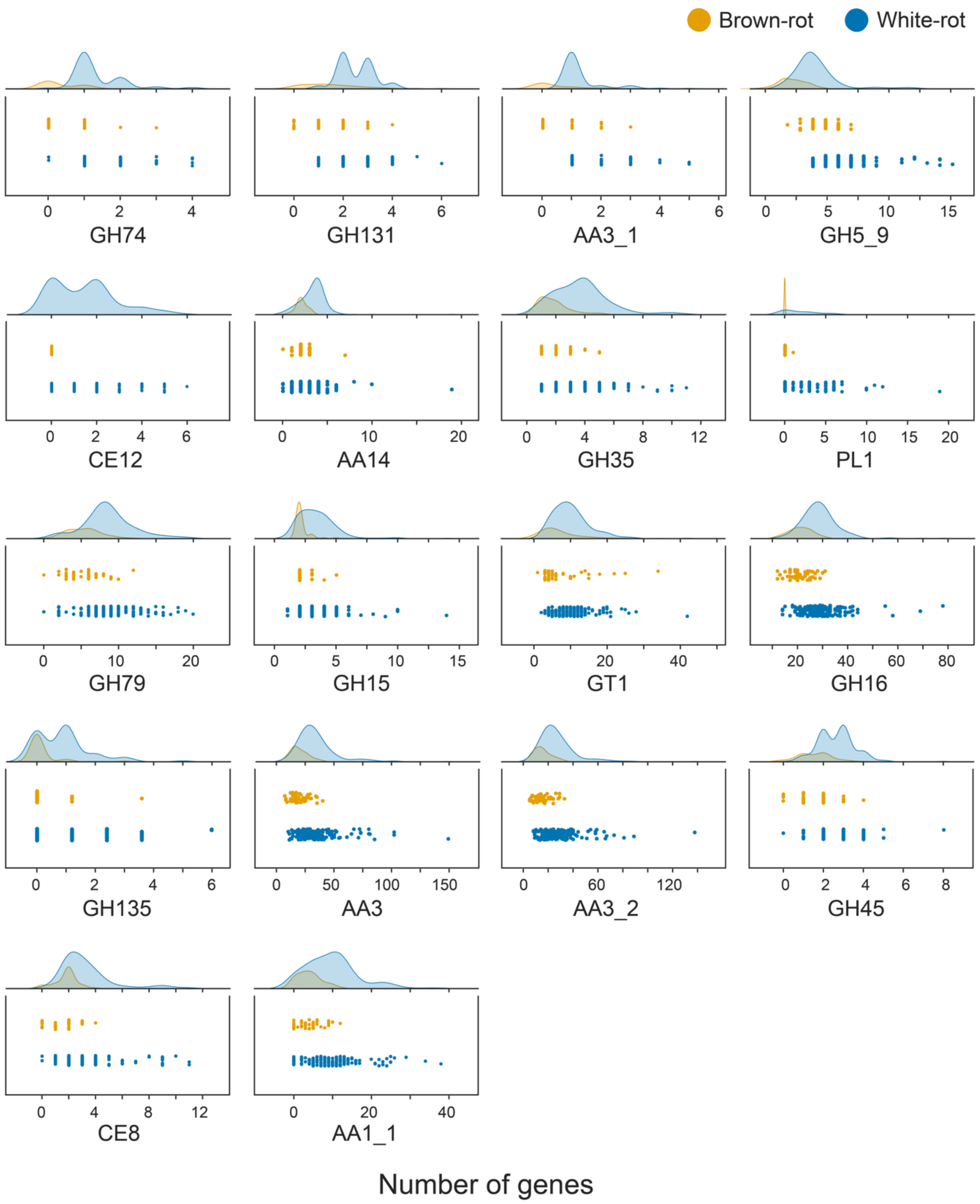

Figure S3 Distribution of genes by decay mode (families ranked 13-30 in importance)

Distribution of the number of genes for families ranked 13-30 in importance in the RF model.

A jitter plot and its kernel density estimation graph are shown.

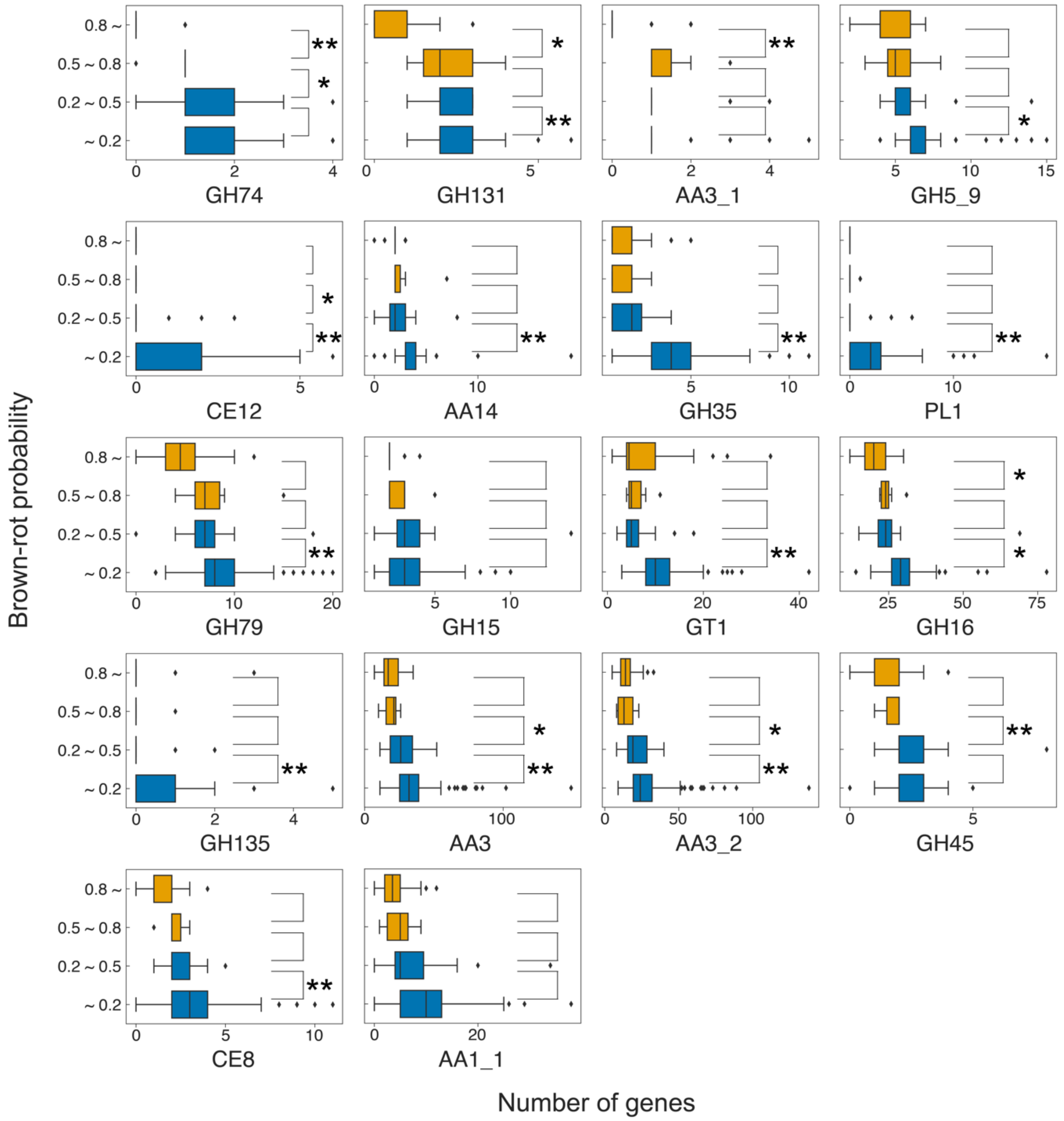

Figure S4 Estimated class probabilities (families ranked 13-30 in importance)

Each sample in the dataset was grouped according to the predicted results of the RF model (class probability estimates of brown rot fungi), and the number of genes in the 13-30 most important CAZy families for each group was shown in a box-and-whisker plot. Welch's t-test was performed between adjacent groups at the 5% significance level (*: $p < 0.05$, **: $p < 0.01$) under the null hypothesis that "there is no difference in gene numbers between the two groups".
